# Effects of Strength Training on Muscle Properties, Physical Function, and Physical Activity among Frail Older People: A Pilot Study

**DOI:** 10.1155/2018/8916274

**Published:** 2018-06-03

**Authors:** Atle Hole Saeterbakken, Hilde Bremseth Bårdstu, Anine Brudeseth, Vidar Andersen

**Affiliations:** Faculty of Teacher Education, Culture and Sport, Western Norway University of Applied Sciences, Bergen, Norway

## Abstract

The aim of this study was to determine the effects of a 10-week strength training intervention on isometric strength, rate of force development (RFD), physical function (stair climbing, rising from a chair, and preferred and maximal walking speed), and physical activity among frail elderly people receiving home-care services. Thirty participants were randomly assigned (by sex) to a control group (CON) or a strength training group (ST) performing a supervised training programme using elastic bands, box-lifting, and body weight exercises twice per week. Twenty-three participants were selected to complete the study (age 84.9 ± 6.1 years). For the ST, only improvement in muscle properties was the peak RFD in leg extension (*p*=0.04). No significant differences were observed in muscle properties for the control group (CON) (*p*=0.16–1.00) or between groups (*p*=0.39–1.00). There were no changes within and between the groups in physical function (*p*=0.12–0.19) or physical activity levels (*p*=0.06–0.73). The results of this pilot study did not demonstrate greater improvements in muscle properties and physical function and improved physical activity after attending a home-based resistance program compared to physical activity advise; however, larger population studies should examine these findings. This trial is registered with ISRCTN10967873.

## 1. Introduction

With increasing age, human skeletal muscle undergoes both structural and functional changes, with a reduction in muscle mass and muscle strength [1, 2]. In addition, ageing atrophy is associated with a notable decrease in maximum muscle strength, power, and rate of force development (RFD) [3, 4]. Reduced muscle mass and muscle strength is associated with loss of function during typical daily living activities such as rising from a chair, stair climbing, and in postural balance [5].

Strength training has improved muscle strength, muscle mass, and physical function among older adults [6–10]. Traditional strength training studies with elderly people are conducted in fitness centres with multiple training machines [6–10]. However, fitness centres may exclude frail older adults to participate due to their functional level, discomfort, lack of confidence, or inability to travel to another place. Further, very few nurse-care centres have well equipped strength training facilities. Recently, the interest in low-cost home-based intervention studies examining the older adults has grown in popularity [11, 12]. A home-based exercise programme may contribute to increased participations, especially among frail older adults who might not have the opportunity to use gym or fitness facilities [13]. However, the isolated effects of home-based strength training programs are not conclusive and have not examined frail older adults [11, 14–17]. Therefore, pilot studies with frail older adults examining training programs (i.e., equipment, volume, frequency, intensity, and ability to participate) need to be conducted before starting large-scale studies.

However, typical equipment used in home-based interventions studies is elastic bands, body weight exercises, and other undersized training devices [13], and the equipment is cheap, portable, and saves space compared to a training centre. Intervention studies for the older adults using this training equipment have found improvements in muscle strength [16, 17]. However, the transferability of the improved strength to physical function (i.e., rising from a chair, stair climbing, and postural balance) is not clear. While traditional and explosive strength training improved RFD by 12–50% among 60–89-year-old participants [18–20], none of the studies examining home-based programs have trained explosive strength or included measurement of RFD.

It is well documented that physical activity (PA) can reduce the risks of chronic diseases and contribute to the enhancement of physical function and maintenance of independence for older adults [21–23]. Overall, the PA level decreases with ageing, and only 12% of the 80–85-year-old age group fulfilled the current PA recommendation of 30 minutes of moderate daily activity [24, 25]. However, there is a lack of knowledge about the effects of low-cost home-based strength training and PA levels among frail older adults. Further, the effects of home-based strength training programs among community-dwelling older adults receiving health-care services have not been properly examined as a rehabilitation strategy to prevent the age-related changes in muscle properties and physical function. With a rapid increasing numbers of older adults in the next centuries, low-cost and home-based rehabilitation programs need to be examined to decrease the factors associated with needy elderly being able to live independent and self-reliant of health-care services. Therefore, the aim of this study was to conduct a pilot study to determine the possible harms or benefits of a home-based strength training programme on physical function tests and level of physical activity in addition to muscle strength and RFD among frail old adults. Accordingly, we hypothesized that a 10-week intervention could be beneficial for frail older adults by improving muscle strength, peak RFD, and physical function, but not the PA due to the short intervention period.

## 2. Materials and Methods

### 2.1. Study Design

The pilot study was parallel group design where 30 community-dwelling older adults receiving health-care services were randomly assigned to either a strength-training group (ST) or a control group (CON). The ST group performed a progressive strength-training programme twice a week with 10–12 repetitions for 10 weeks. The CON was instructed and encouraged to continue their normal activities during the intervention. Pre- and postintervention, all participants were tested in maximal and explosive strength and physical function tests (walking speed (preferred and maximal), stair climbing, and rising from a chair) and wore an accelerometer for six days to determine the physical activity.

### 2.2. Participants

**Figure 1:**
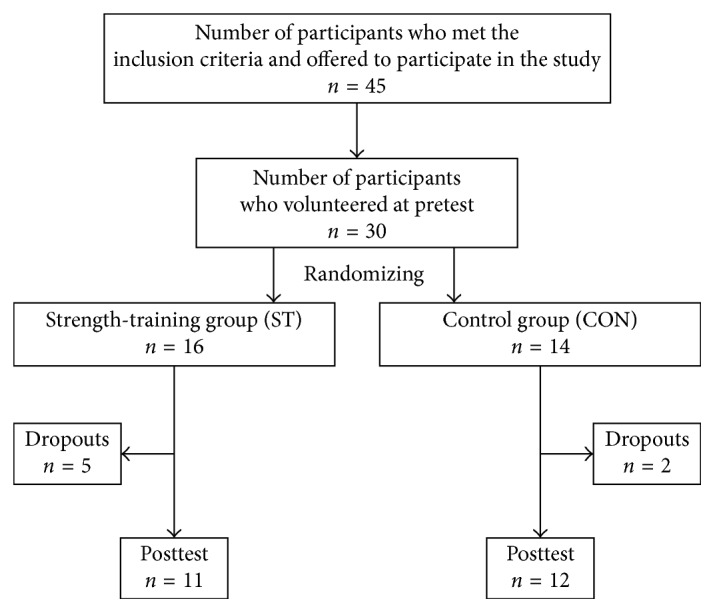
An overview of the study design.

Due to low founding, only one municipality was invited to participate in this pilot study. The health-care services in a municipality (7000 residents) informed all elderly people meeting the inclusion criteria (45 potential participants) to participate in this pilot study. 2/3 of them volunteered, and 30 community-dwelling older adults (6 male and 24 female) were recruited. The participants were stratified randomly by sex. The name of each participant was written on a patch and divided into two heaps—one for each sex. The patches were thoroughly mixed before drawing first the male and then the female to either a strength-training group (ST) (*n*=16) or a control group (CON) (*n*=14). The first, third, fifth, and more patches from each heaps were assigned to the ST group and remaining to the CON group. Seven participants dropped out during the intervention (five from the ST and two from the CON) with reason not related to the study. Twenty-three participants (6 male and 17 female) aged 71–97 years completed the study (Figure 1). Due to the nature of the intervention, neither participants nor staff were blinded to the group allocation or the outcome assessors. Anthropometric measurements for the two groups are shown in Table 1.

To participate in the study, the following inclusion criteria had to be fulfilled: (1) >70 years of age living at home and (2) in need of home care due to functional disabilities and/or medication. The exclusion criteria were older adults diagnosed with chronic mental disorders, for example, Alzheimer's disease or injuries and/or diagnoses not justifiable to execute testing or training. Explanation of the purpose, procedures, potential risks, and benefits of the study was given orally and in writing to all participants. A written consent was provided prior to testing. The study was approved by the local ethics committee (REK sør-øst B, 2014/1147).

### 2.3. Measures

#### 2.3.1. Maximal Strength and Rate of Force Development (RFD)

To test the maximal isometric force output in leg extension and elbow flexion, a nonelectric sling (ROPES A/S, Asgardstrand, Norway) was placed around the dominant ankle or hand and attached to a force cell (Ergotest A/S, Porsgrunn, Norway). The knee and elbow angles were measured for each participant with a paragraph protractor. Both tests were performed with a 90° angle flexion in the knee or elbow joint. Performing the leg extension test, the participants were seated with a hip angle of 90° which had to be maintained during testing [15]. The participants' dominant hand was used to holda semisupinated grip [26], and the elbow was held closed to the body. Each participant had three attempts with a contraction duration of five seconds and a 60-second rest between each attempt [27]. Participants were carefully instructed to contract “as fast and forcefully as possible.” The highest force output over a three seconds window was used in the analysis. The rate of force development (RDF) was calculated over a 200-millisecond sampling window where the steepest vertical generation occurred [19].

#### 2.3.2. Physical Performance Tests

Four physical performance tests, designed to replicate daily living activities, were conducted [14, 28, 29]. All tests were performed minimum twice and maximum three times based on the physical capacity of the participants. The best attempt was used for further analysis. Participants who had problems executing the tests used necessary support, that is, an aid for walking such as a stick or crutches during the walking test, handrails for stair climbing, or armrests for rising from a chair. The use of support and number of attempts by each participant were noted for similar execution at posttest. 72% and 49% of the participants in the ST and the CON, respectively, needed support to perform one or more tests.

Two measurements of walking speed were carried out: preferred walking speed, in which the participants were instructed to walk at a pace similar to daily walking speed, and maximal speed, where the participants walked as fast as they could without running. The time was assessed by photocells (Ergotest A/S, Porsgrunn, Norway) placed 20 meters apart along a corridor. The first photocell was placed 2 metres in front of the start line for proper acceleration.

In the stair climbing test, the participants were instructed to ascend a staircase consisting of 16 steps with an 8 cm rise per step as fast as possible. Time was assessed using photocells (Ergotest A/S, Porsgrunn, Norway) placed at the beginning and end of the staircase. The staircase had a handrail on each side, and the participants were instructed to perform the test in the same way as they normally ascended a staircase.

In the rising from a chair test, the participants were seated in a hard-backed chair (seat height 44 cm from the floor) with their arms folded across their chest. The participants were instructed to rise to a full-standing position and return to a full-sitting position five times as fast as possible. The time was assessed using a stopwatch. The participants who were not able to rise without the use of aids (walker or armrests) were allowed to use the aids.

#### 2.3.3. Physical Activity

Physical activity (PA) was measured using an accelerometer (ActiGraph GT1M, ActiGraph, LLC, Pensacola, Florida, USA) before and after the intervention. The measures were carried out over three weekdays and the weekend (Wednesday to Sunday). Valid registrations had to represent a minimum of eight hours of valid registration per day with three approved days to be included in the analysis [30]. The participants were instructed to wear the accelerometer on the right hip while awake and to remove the accelerometer only during night time and during water activities. All registrations between 12:00 am and 06:00 am were excluded [25]. Nonwear time was defined by an interval of at least 60 consecutive minutes of zero activity intensity counts, with allowances of 1-2 minutes of counts between 1 and 100 [30]. Based on previous studies, a 10-second epoch period was used [31]. Adult standard was used for overall physical activity level (counts per minute) in addition to limits for three different intensity zones. The intensity threshold criteria were 100–2019 counts for low intensity, 2020–5999 for moderate intensity, and over 5999 counts for vigorous intensity. Activity under 100 counts per minute was registered as inactivity [30]. The number of steps per day was registered using an embedded pedometer function [25]. The software program Actilife v 6.10.1 (Actigraph, LLC, Pensacola, Florida, USA) was used for options and analyses.

#### 2.3.4. Intervention

The CON group was encouraged and instructed to continue their normal activities, to stay physically active, and to make physically active choices. An individual conversation between the participants and a health professional regarding the importance of staying physically active, make physically active choices, and the benefits of physical activity was conducted for the CON group. The session was conducted at the beginning of the intervention period. The conversation took place in the residence of each participant in the CON group and lasted between 30 and 45 minutes. In addition, they received a folder from the health ministry with information, benefits, and recommendations of physical activity.

The participants in the strength-training group performed a progressive strength-training programme twice a week for 10 weeks. The participants were instructed to perform 10–12 repetitions maximum at a controlled tempo but concentrating on a fast explosive concentric phase and a slow eccentric phase [29]. A professional training instructor was present in every training session to make sure of the correct technique, intensity, and numbers of sets. The training load gradually increased in numbers of sets and resistance level. The participants were instructed to add greater resistance when they could easily perform 10–12 repetitions of a movement in the last set without significant fatigue (i.e., perform 5 extra repetitions). One–four weeks of training consisted of two sets per exercise. The number of sets was increased to three sets from week five and throughout the intervention. The training sessions were performed with a training instructor present. The participants had to attend minimum 80% of the sessions for a valid training quantity. The mean training attendance was 84%.

**Figure 2:**
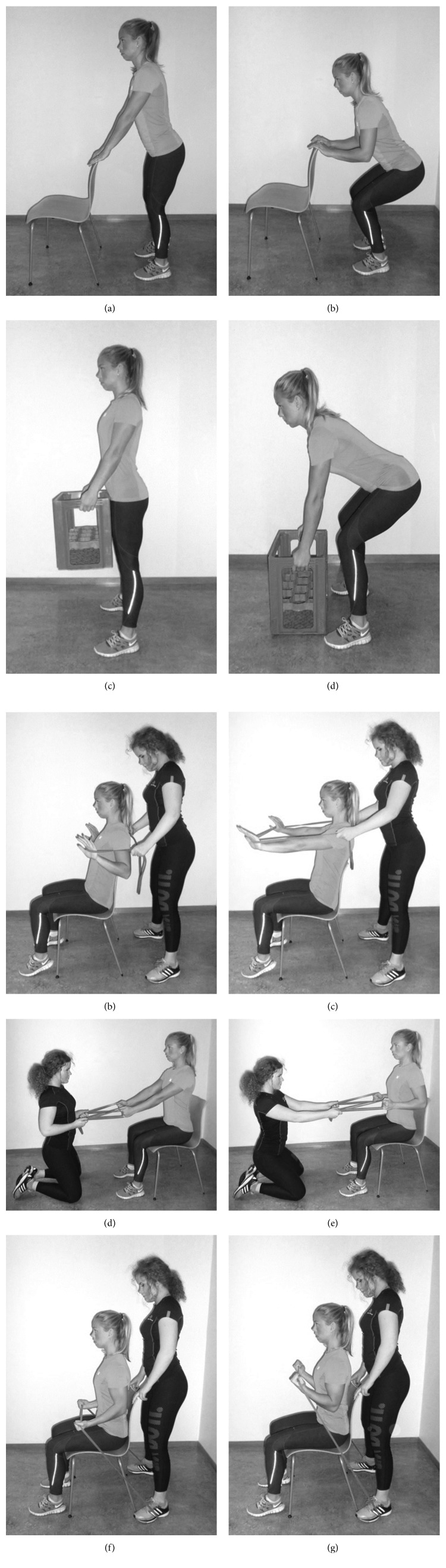
(a–j) An overview of the five training exercises.

The programme consisted of five minutes of general warmup and 40–55 minutes of strength training depending on the number of sets. Five exercises were conducted: squats, box lifts, seated row, chest press, and biceps curl. Squats were performed using the body weight as resistance (Figures 2(a) and 2(b)). Box lifts were performed with a soda crate as resistance (Figures 2(c) and 2(d)). The external weight was gradually increased by placing 0.5 or 1 litre bottles of water in the crate. Elastic bands were used as resistance in the exercises seated row, chest press, and biceps curl. The training instructor held the elastic band for the chest and rowing exercises (Figures 2(e)–2(h)). Biceps curls were performed seated by placing the elastic band underneath the feet of the training instructor with an equal length in each hand (Figures 2(i) and 2(j)). Further, three different elastic bands of assorted colours were used, with each colour denoting a different resistance level [32]. The bands required a force of approximately 79, 181, and 283 Newton, respectively, to stretch the bands to double their length.

#### 2.3.5. Data Analysis

To assess differences in physical function, muscle strength, and physical activity, we used a two-way (groups × time) within-between analysis of variance (ANOVA) with repeated measures. When a significant interaction was detected by ANOVA, paired *t*-tests with Bonferroni post hoc correction were applied to locate where the differences lay. Tests were analysed using the SPSS (SPSS 23; SPSS Inc., Chicago, IL USA) statistical software package and were analysed per protocol. All results are presented as mean ± SD unless otherwise noted, and significant results are presented with a Cohen's *d* effect size (ES) of 0.2 considered small, 0.5 medium, and 0.8 large [33]. A *p* level of 0.05 was used for statistical significance.

## 3. Results

### 3.1. Maximal Strength and Rate of Force Development (RFD)

**Figure 3:**
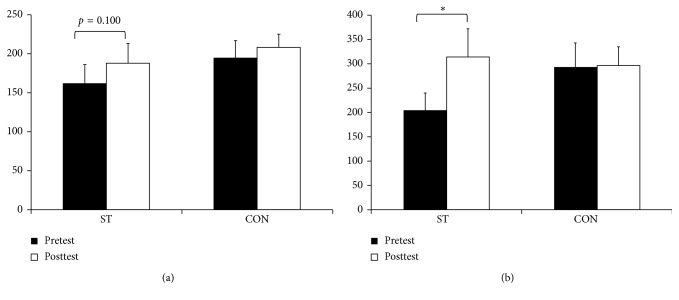
The pre- and posttest results of the (a) maximal strength and (b) RFD in leg extension for the strength-training group (ST) and control group (CON). All values are presented as mean ± SE. ^*∗*^Within-group differences, *p* < 0.05.

**Figure 4:**
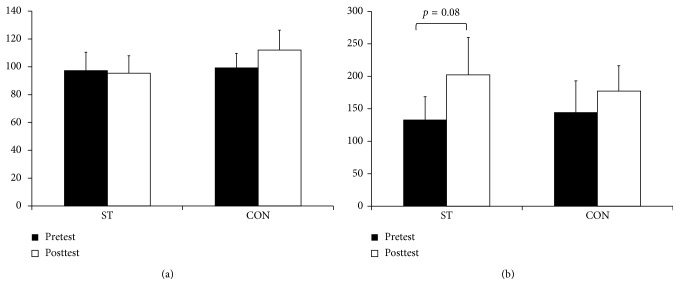
The pre- and posttest results of the (a) maximal strength and (b) RFD in arm flexion for the strength-training group (ST) and control group (CON). All values are presented as mean ± SE. ^*∗*^Within-group differences, *p* < 0.05.

For the maximal strength and RFD, there was no interaction for the (*F*=0.714–4.114, *p*=0.0.3–0.409) or a main effect for groups (*F*=0.032–0.269, *p*=0.390–0.861). With exceptions for maximal strength in the arm (*F*=1.162, *p*=0.294), there was a main effect for time (*F*=4.473–10.043, *p*=0.005–0.047). After the post hoc tests, the ST group had a 15.3% nonsignificant improvement in the leg extension test (*p*=0.10, ES=0.31) and a 53.1% increase for peak RFD (*p*=0.04, ES=0.69). In the elbow flexion test, a nonsignificant 51.3% improvement for peak RFD (*p*=0.08, ES=0.60) was observed. No significant differences were observed for the CON (*p*=0.16–1.00) (Figures 3 and 4).

### 3.2. Physical Function

For the physical function tests, there was no interaction (*F*=0.087–2.519, *p*=0.130–0.770) or a main effect for groups (*F*=1.888–2.696, *p*=0.115–0.186) or time (*F*=0.55–2.223, *p*=0.151–0.817) with one exception. The stair climbing test had significant main effect for time (*F*=4.659, *p*=0.044). Despite an overall improvement in stair climbing of 26.6%, the improvement was not significant for the ST (*p*=0.22). Furthermore, no significant difference was observed for the CON (*p*=0.22). All details are presented in Table 2.

### 3.3. Physical Activity (PA)

For PA, there was no interaction (*F*=0.009–0.939, *p*=0.341–0.927), main effects for time (0.001–0.223, *p*=0.644–0.980) or group (*F*=0.120–4.178, *p*=0.059–0.734) for the variables overall PA, inactivity, moderate intensity, high intensity, and numbers of steps per day. However, an interaction was observed for the variable low intensity (*F*=5.008, *p*=0.041). Post hoc tests demonstrated no differences between the groups (*p*=0.480–1.000) or differences between pre- and posttest (*p*=0.133–0.698). All details are presented in Table 3.

## 4. Discussion

The home-based strength-training intervention among frail older adults did not increase the strength but improved RFD in the lower body for the strength-training group. Furthermore, there were no significant changes in physical function, and the physical activity level was unaltered after the intervention. No differences were observed in the CON group or between the groups in any of the tests.

Leg extension strength increased by 15.3% in the ST but only tended towards statistical significant (*p*=0.100). None of the participants trained strength before the intervention. The numbers of exercises and sets were therefore low. Additionally, the strength-training group only trained twice per week. The lack of improvement was most likely a result of low training volume, low statistical power, and large standard deviation. The result supports not our hypotheses nor previous studies despite similar improvements as comparable studies [16, 17, 34]. For example, Capodaglio et al. demonstrated a 14.9% improvement in maximal isometric leg extension after five months of strength training performed at home using elastic bands [34]. In addition, Frontera et al. observed an 11.9% increase in the right quadriceps cross-section area in addition to an 8.5% improvement in dynamic muscle strength after attending a 12 wk strength training program [35].

The arm flexion strength was unaltered for the ST. Despite the participants' effort to perform the exercises with proper execution until failure, the limited training load on the biceps (only one isolating exercise) may explain the lack of strength improvement. Furthermore, the moderate training load using an elastic band and the choice of 10–12 repetitions in the present study may have contributed to the lack of improvement in upper-body strength. However, the number of repetitions is recommended for novice individuals [36]. Still, the same findings were presented by Skelton and McLaughlin, who failed to observe an improvement in isometric elbow flexion despite a 20% improvement in leg extension after an eight-week supervised training period among 80-year-olds [5]. Furthermore, Zion et al. used a similar training protocol as the present study with only one isolating exercise for biceps and observed no changes in isometric handgrip [12]. Importantly, the training in the present study was performed dynamically while the tests were isometric. Previous studies have reported substantially lower improvement in isometric than dynamic strength after a dynamic strength-training program [37–39].

Our findings demonstrate that the execution of the exercises, performed in a fast explosive concentric phase, contributed to the improvements in RFD. These results support our hypotheses and previous studies [18–20, 40]. The increase in RFD in the present study may be the result of neurological adaptations as RFD is highly influenced by the magnitude of neuromuscular activity irrespective of age [41, 42]. To our knowledge, no previous studies have carried out RFD tests after home-based strength training using low costs portable training equipment. Previous studies have used traditional strength training equipment (training machines or free weights), but the need of such facilities may exclude several frail older adults. Comparing the strength and RFD results, the substantially lower strength improvement is supported by previous studies. The improvement in RFD has shown to increase greater than isometric strength [40], and accordingly, there is evidence that the decline in muscle power is greater than the decline in muscle strength in the older adults [3, 43]. Older adults may therefore have a larger potential to improve the RFD compared to muscle strength.

This study did not demonstrate a significant effect of the resistance training programme on functional outcome measures. However, there was a consistent trend towards nonsignificant improvements in physical function by 3.5–25.6% for the ST. Although the training programme trained the same muscles/muscles groups as the physical tests target, the exercises might not have been specific enough to give significant changes in the physical functional tests [38]. Moreover, the absence of significant changes in physical function may be explained by variability in performance, age (78–97 years), and physical characteristics at baseline. For example, differences in execution of the tests due to frailty and the need for support (i.e., walking aids and the use of handrails and armrests) may have influenced the variation in the test results within and between groups. However, the older adults with assistive devices (3 in the ST and 4 in the CON) were not significant differences in the physical function tests compared to the one without.

Comparable studies have usually carried out the tests with identical execution for all participants [11, 29, 44], and this might contribute to less variation in the test results, mainly because the participants in these studies were younger and healthier, resulting in more homogeneity compared to the participants in our study. Despite the differences in execution among the participants, the test protocol was carefully standardized and the use of support was noted for similar execution at pre- and posttest. The training volume was quite low (2x per week and only 2 sets in the first weeks) due to the lack of experience with resistance training and relative low physical function (all participants received health-care services). We cannot exclude that a higher volume may have resulted in greater benefits.

Our findings are supported by a previous study who reported no significant changes, but a tendency for improvements in rising from a chair (10 times) and the time-up and go among frail older adults after resistance training intervention [45].

The physical activity level concerning inactivity and low, moderate, and high intensity was unaltered for both groups after the intervention. However, the overall PA (counts per minute) increased with 17.4% for the ST, but not significantly. Importantly, the ST maintained their physical activity attending strength training twice per week. A systematic review demonstrated a compensation of physical activity in over 50% of the studies attending different training interventions [46]. Despite having benefits of participation in the strength-training program, the physical activity level of the ST was not increased. The physical activity results supported our hypothesis that due the short intervention period, the PA level did not increase. Importantly and most likely, our findings may have been affected by the variation of the season, with pretesting being performed in late summer and posttesting during the late autumn. In addition, the weather conditions may have influenced the activity level due to dry weather at pretest and precipitation/snow at posttest and might explain the nonsignificant changes. Lemmer et al. demonstrated no significant changes in PA for younger (20–30 years) or older adult (65–75 years) individuals after 24 weeks of strength training supporting the PA results of the present study [47].

With a small sample size, there is a risk of conducting type II errors and the additional moderate dropout rate of five participants in the ST and two in the CON. The results should therefore be interpreted with caution. Importantly, the participants were frail older adults, but we experienced no harms of unintended effects. The only harms observed were delayed onset of muscle soreness but only in the first weeks. Furthermore, several of the participants in the strength-training group reported different and often several muscle-skeletal disorders. Still, none reported greater pain after the intervention, but rather the opposite. There are some limitations to the current study that need to be addressed. There was a large variation in physical performance, age, and physical characteristics (i.e., need of walking aid). The large variation may therefore influence the statistical analyses. To reduce a possible learning effect, we intended to complete two testing sessions as part of the baseline measures. Unfortunately, only one session of testing was performed at baseline due to a time limitation for the intervention. Moreover, the lack of statistically significant findings in physical function and the PA level may be related to the high variability of the results and the differences in weather conditions.

Further studies should include a more homogeneous population (i.e., not need of walking aid), decrease the variation in age (and thereby the age related loss of muscle mass), and recruit more participants. Still, a larger population may detect differences among older adults with need of walking aids and those without as well as differences between age groups (i.e., 70–80 years versus 80–90 years). Including older adults with receiving health-care services, it is important to gradually increase intensity, sets, and training frequency. Long-lasting intervention studies should therefore try to increase the training volume to a greater extent than the present. Finally, further studies should include factor analyses with age and assistive devices as two important factors, as well as analyses of the ability to live self-reliant and independent of further health-care services.

## 5. Conclusion

The results of this pilot study did not demonstrate greater improvement in muscle properties and physical function and improved physical activity after attending a home-based resistance program compared to physical activity advice. However, the strength-training group improved explosive strength. Hopefully, further trails would benefit from this pilot study and make rehabilitation strategies with older adults a research priority.

## Figures and Tables

**Table 1:** Age, height, and BMI for both groups.

	ST (*n*=11)	CON (*n*=12)	*p* value between groups
Age (years)	86.5 ± 6.4	83.5 ± 5.7	0.26
Height (cm)	163.1 ± 9.5	165.4 ± 11.2	0.73
Weight (kg)	64.3 ± 21.2	66.6 ± 8.7	0.60
BMI (kg/m^2^)	23.9 ± 6.2	24.3 ± 1.6	0.81

ST = strength-training group; CON = control group; cm = centimetres; kg = kilograms; BMI = body mass index; all values are presented as mean ±standard deviation.

**Table 2:** Pre- and posttest results of physical function for the ST and CON.

Tests	Group	Pretest	Posttest
Stair climbing (sec)	ST	26.0 ± 6.0	19.3 ± 3.4
	CON	18.3 ± 3.6	17.1 ± 3.3

Preferred walking speed (km/h)	ST	2.3 ± 0.3	2.3 ± 0.2
	CON	3.0 ± 0.3	2.9 ± 0.3

Maximal walking speed (km/h)	ST	3.5 ± 0.3	3.8 ± 0.4
	CON	4.5 ± 0.4	4.3 ± 0.4

Rising from a chair (sec)	ST	27.6 ± 4.6	25.5 ± 4.1
	CON	20.6 ± 2.2	19.1 ± 2.3

ST = strength-training group; CON = control group; sec = seconds; km/h =kilometres per hour; all values are presented as mean ± standard error.

**Table 3:** Pre- and posttest results of physical activity for the ST and CON.

Tests	Group	Pretest	Posttest
Overall PA (cpm)	ST	57.6 ± 8.4	67.7 ± 12.5
	CON	129.5 ± 25.5	120.5 ± 24.7

Inactivity (min/day)	ST	673.4 ± 16.3	688.0 ± 32.6
	CON	663.1 ± 28.4	621.7 ± 31.2

Low intensity (min/day)	ST	83.8 ± 12.8	91.1 ± 13.0
	CON	120.4 ± 21.5	101.6 ± 16.4

Moderate intensity (min/day)	ST	2.9 ± 0.5	2.8 ± 0.7
	CON	11.1 ± 2.8	10.7 ± 3.7

High intensity (min/day)	ST	0.2 ± 0.1	0.2 ± 0.1
	CON	0.2 ± 0.1	0.2 ± 0.1

Steps (per day)	ST	1360 ± 322	1262 ± 312
	CON	2868 ± 547	2396 ± 501

ST = strength-training group; CON = control group; s = seconds; km/h =kilometres per hour; cpm = counts per minute; min/day = minutes per day; all values are presented as mean ± standard error.
